# Early intestinal *Bacteroides fragilis *colonisation and development of asthma

**DOI:** 10.1186/1471-2466-8-19

**Published:** 2008-09-26

**Authors:** Carl Vael, Vera Nelen, Stijn L Verhulst, Herman Goossens, Kristine N Desager

**Affiliations:** 1 Department of Microbiology, University of Antwerp, Antwerp, Belgium; 2 Department of Health, Provincial Institute for Hygiene, Antwerp, Belgium; 3 Department of Pediatrics, University of Antwerp, Antwerp, Belgium

## Abstract

**Background:**

The 'hygiene hypothesis' suggests that early exposure to microbes can be protective against atopic disease. The intestinal microbial flora could operate as an important postnatal regulator of the Th1/Th2 balance. The aim of the study was to investigate the association between early intestinal colonisation and the development of asthma in the first 3 years of life.

**Methods:**

In a prospective birth cohort, 117 children were classified according to the Asthma Predictive Index. A positive index included wheezing during the first three years of life combined with eczema in the child in the first years of life or with a parental history of asthma. A faecal sample was taken at the age of 3 weeks and cultured on selective media.

**Results:**

Asthma Predictive Index was positive in 26/117 (22%) of the children. The prevalence of colonisation with *Bacteroides fragilis *was higher at 3 weeks in index+ compared to index- children (64% vs. 34% p < 0,05). *Bacteroides fragilis *and *Total Anaerobes *counts at 3 weeks were significantly higher in children with a positive index as compared with those without. After adjusting for confounders a positive association was found between *Bacteroides fragilis *colonisation and Asthma Predictive Index (odds ratio: 4,4; confidence interval: 1,7 – 11,8).

**Conclusion:**

*Bacteroides fragilis *colonisation at age 3 weeks is an early indicator of possible asthma later in life. This study could provide the means for more accurate targeting of treatment and prevention and thus more effective and better controlled modulation of the microbial milieu.

## Background

Wheezing at an early age is a major health problem: at least 1 episode of wheezing occurs in 50% of children under 5 years [1]. Wheezing children are a heterogeneous group and many patterns of wheezing disorders seem to exist: some children wheeze with viral infections only during the first years of life and do not subsequently develop asthma and some may have early childhood asthma. Studies of the natural history of the disease have demonstrated that in most cases of persistent asthma the initial asthma-like symptoms occur during the first several years of life.

The mechanism by which predisposing and protective risk factors interact to induce asthma remains unclear. The 'hygiene hypothesis' suggests that early exposure to microbes and allergens can be protective: a western lifestyle causes the immune system to mature in a way that predisposes to asthma in genetically prone individuals [2]. The intestinal microbial flora could operate as an important postnatal regulator of the Th1 and Th2 balance. The normal intestinal flora is essential in the maintenance of oral immune tolerance by inhibition of potential lymphocyte reactivity [3]. Specific bacterial surface determinants and DNA sequences induce production of Th1 cytokines through NF-kB and STAT signaling pathways in human macrophages. *Lactobacillus rhamnosus *"GG" has been shown to induce this response [4]. The gut flora of healthy infants shows differences with allergic infants. More *Clostridia *and fewer *Bifidobacteria *were found in infants at 3 weeks and 3 months of age who would later develop atopy [5]. In allergic infants (2–7 months of age) more *Bifidobacterium adolescentis *and *longum *were found as compared to healthy infants showing more *Bifidobacterium bifidum *[6].

Large scale epidemiological studies studying the microbial gut flora in young children from the general population are lacking.

The Asthma and Allergy study evaluates the intestinal flora in a prospective birth cohort recruited from the general population and followed up to the age of 3 years. A total of 158 children were included, the largest cohort ever that studied the relation between microbial gut flora and asthma. A positive association was found between early *Bacteroides fragilis *colonisation and a positive Asthma Predictive Index, a clinical predictor of school age asthma.

## Methods

The Asthma and Allergy study is a prospective birth cohort and part of the Environmental Health action of the Flemish Ministry of Health and Environment. Children (n = 158) were recruited through maternity clinics in Flanders. Selection criteria for enrolment in the study were vaginal delivery at term and uncomplicated perinatal period. Questionnaires were collected with data on the parents, including demography, smoking and asthma. Data of the child on demography, respiratory symptoms and risk factors for asthma were collected by postal questionnaires send every 6 months starting at the age of 3 weeks until the age of 36 months. The question on the presence of wheezing referred to the period between two questionnaires, e.g. the presence of wheezing in the questionnaire at 6 months referred to the time period between 3 weeks and 6 months. The study protocol was approved by the medical ethics committees of the participating institutes. All parents gave written informed consent.

Symptoms of wheeze were assessed by International Study of Asthma and Allergies in Childhood core questions [7]. Information about doctor's diagnosed parental asthma was collected by the following question: "Did a doctor ever diagnose asthma?". Based on the longitudinal questionnaire data on wheeze symptoms in the first 3 years of life, children were classified according to the 'loose' Asthma Predictive Index (API) into an API positive and an API negative group. According to the 'loose' index a positive API included wheezing during the first three years of life and eczema or parental history of asthma [8].

Approximately 2 g of stools was collected into a sterile recipient by the parents at 3 weeks of age. The sample was sent to the laboratory under anaerobic conditions where it was stored immediately at -70°C until analysis. A total anaerobe count was obtained by quantitative plating of a saline suspension of faeces on Columbia blood agar after 4 days of anaerobic incubation at 37°C. The saline suspension of faeces consisted of 1 g of wet faeces diluted in 10 ml of sterile saline solution, homogenized using a stomacher. Selective media were used to study the bacterial subpopulations [9]. *Bacteroides fragilis *group (subsequently called *Bacteroides fragilis*) was determined quantitatively on Bacteroides Bile Esculin agar (BBE) (Becton-Dickinson, Belgium); only black pigmented colonies obtained after 4 days of anaerobic incubation at 37°C were considered [10]. *Bifidobacterium *was determined on mupirocin (100 μg/ml) trypticase-phytone-yeast agar; only colonies smaller than 0.7 mm obtained after 4 days of anaerobic incubation at 37°C were considered [11]. *Lactobacilus *was cultured on LAMVAB medium [12]. Green and white colonies obtained after 4 days of anaerobic incubation were considered. *Clostridium *counts were obtained after pretreatment of the faecal sample in 80% ethanol for 15 min. and subsequent culture on Columbia blood agar after 4 days of anaerobic incubation at 37°C.

Because bacterial counts followed a right-skewed distribution, data were log10-transformed. Chi-square was used to compare characteristics of API positive versus API negative subjects. The not normally distributed log transformed counts were compared using non-parametric tests (Friedman and Mann-Whitney rank sum test where appropriate using SPSS software version 12.0). Median and range of the bacterial counts were reported as log _10 _CFU/g faeces. The detection level was ≥ 3 log CFU/g. *Bacteroides fragilis *group colonisation was estimated by multiple logistic regression analysis, with colonisation as the independent variable and asthma predictive index as dependent variables. We applied multiple imputation to investigate the stability of the results due to missing data. For multiple imputations, each missing value was replaced with a set of numbers (Markov chain Monte Carlo method) [13]. We analysed the results of each imputation as complete data sets and we combined the results of these analyses.

## Results

### Description of the study population

Of the 158 children recruited in this study, 54% were boys. Maternal or paternal asthma was present in 8% and 5% of the children, respectively. Several children were lost for follow-up at the end of the 3 year study period. As a result, API at age 3 years could not be determined in 41 of the 158 children due to missing data on wheezing (n = 30) or on eczema (n = 9) of the child in the 6 monthly questionnaires or on parental asthma (n = 5). There were no differences in the percentage of children with wheeze at any age, parental asthma, and eczema at any age or gender of the infant between children who could or could not be categorized according to API. API was positive in 26/117 (22%) of the children. Table 1 describes the characteristics of the API+ and API- children.

**Table 1:** Characteristics for API+ versus API- children at age 3 years.

Factor	API +	API-	P value
Gender (Male)	18/26 (69%)	48/91 (53%)	NS
Exclusive breast feeding ≤ 3 weeks	20/26 (77%)	63/91 (69%)	NS
Antibiotic use ≤ 3 weeks	0/26 (0%)	2/90 (2%)	NS
Wheeze ever	26/26 (100%)	41/91 (45%)	< 0,0001
Eczema ever	20/26 (77%)	9/90 (10%)	< 0,0001
Maternal asthma	6/25 (24%)	3/90 (3%)	0,003
Paternal asthma	6/23 (26%)	1/88 (1%)	< 0,0001
Maternal smoking during pregnancy	2/26 (8%)	5/89 (6%)	NS

### Faecal microorganisms in the study population

A total of 149 faecal samples were collected, which is a response rate of 94%. The prevalence of colonisation with *Bacteroides fragilis *was higher at 3 weeks in API+ children than in API- children (64% vs. 34%, p < 0. 05, table 2). *Bacteroides fragilis *and *Total Anaerobes *counts at 3 weeks, expressed in log CFU/g, were significantly higher in children with positive API as compared with those without (table 3).

**Table 2:** Prevalence of faecal microorganisms at 3 weeks of age

Bacteria	API- (n = 91)	API+ (n = 26)
*Bacteroides fragilis*	34*	64*
*Bifidobacterium*	91	92
*Lactobacillus*	37	32
*Clostridium*	57	60
*Staphylococcus*	89	76
*Total Anaerobes*	99	100

Prevalence (%) of microorganisms for API+ versus API- (n = number of infants): *: p < 0.05

**Table 3:** Median counts of faecal microorganisms (log CFU/g) (range) in API- and API+ children at the age of 3 weeks

Bacteria	API- (n = 91)	API+ (n = 26)
*Bacteroides fragilis*	1,8* (0–8,7)	3,5* (0–8,2)
*Bifidobacterium*	5,6 (0–9,1)	5,8 (0–8,7)
*Lactobacillus*	2,1 (0–8,6)	1,7 (0–8,5)
*Clostridium*	2,7 (0–6,2)	2,8 (0–6,1)
*Staphylococcus*	4,4 (0–8,6)	3,9 (0–8,1)
*Total Anaerobes*	7,3* (0–9,1)	7,9* (6,3–8,9)

Counts of faecal microorganisms for API+ versus API- (n = number of infants): *:p < 0.05.

### Logistic regression analysis

There were no major differences between the results of the crude and adjusted analyses for *Bacteroides fragilis *colonisation. Further adjustment for exclusive breast feeding, maternal smoking during pregnancy, infant use of antibiotics at age of 3 weeks, parental socio-economic status and gender did not alter the results of the analysis (table 4).

**Table 4:** Multiple logistic regression analysis of risk factors for outcome variable Asthma Predictive Index at age 3 years

Variable	OR	CI	P
Colonisation with *Bacteroides fragilis *at the age of 3 weeks (yes versus no)	4,4	1,7 – 11,8	0,003
Exclusive breastfeeding age below the age of 3 weeks (yes versus no)	2,0	0,6 – 6,1	0,250
Maternal smoking during pregnancy (yes versus no)	2,1	0,3 – 14,1	0,436
Infant use of antibiotics below the age of 3 weeks (yes versus no)	7,6	0,4 – 157,6	0,189
Parental socioeconomic status (low versus high)	0,9	0,3 – 2,4	0,825
Infant gender (male versus female)	0,6	0,2 – 1,7	0,367

Odds ratios (95% confidence interval) are adjusted for the independent variables, indicated in the rows of the table.

OR: adjusted odds ratio, CI: 95% confidence interval

### Multiple imputation analysis

Colonisation with *Bacteroides fragilis *was still significantly associated with API both univariately (odds ratio = 3.57; p = 0.002) and in a multiple model controlling for the factors in Table 4 (odds ratio = 2.75; p = 0.01).

## Discussion

This is the first prospective cohort study that links gut flora to API (a clinically relevant risk factor for developing asthma) at the age of 3 years. It was demonstrated that differences in gut flora precede the development of asthma: early colonisation with *Bacteroides fragilis *was more often found in recurrent wheezing children at risk of persistent asthma at school age. Differences in feeding or use of antibiotics cannot explain the findings.

Between 25 and 50% of children suffer from one or more wheezing episodes below the age of 6. However, wheezing illness below the age of 6 years is not a single disease, but comprises several distinct disorders. Asthma phenotypes have been identified in epidemiologic trials, each with their own risk factors and different outcome. Overall, 30–40% of the children presenting with wheezing below the age of 3, will wheeze later in life. Decreased airway calibre could be an important factor in pathophysiology, explaining the transient nature of wheeze in many children. The diagnosis is not straightforward since no simple clinical tools are available to discriminate children prone to develop persistent asthma from those who will not. The 'Asthma Prediction Index' has been associated with an increased risk for asthma at school age [8]. This index was chosen as outcome parameter for the study since it is at present the best tool for prediction of asthma at school age. A 'stringent' index that required subjects to have wheezed more frequently in early life combined with risk factors for asthma had an acceptable positive predictive value (e.g. at age 13: 51%) and a very high specificity (97%), but its sensitivity was quite low (15%). Conversely, a more 'loose' index, which only required infrequent wheezing episodes plus the same combination of other risk factors included in the 'stringent' index had a much higher sensitivity (39%) but lower specificity (82%) and positive predictive values (32%). The negative predictive value at all ages was very high for both indices, suggesting that the great majority of children who did not develop asthma during the school years had a negative predicted index during the first years of life. Because the Asthma Predictive Index is only an approximation to predict which children will subsequently develop persistent asthma, further follow-up at school age is required to definitely determine the relation between early *Bacteroides fragilis *colonisation and asthma.

At birth the gastrointestinal tract is sterile and becomes colonised during the first months of life until an adult-pattern of stable gut flora is established. The gut associated lymphoid tissue (GALT) matures during the first year of life, which requires microbial stimulation. Without such stimuli inappropriate GALT maturation results in defects in the oral tolerance [14]. Germ-free mice could develop oral tolerance if the gut flora was reconstituted with *Bifidobacterium *not later than during the neonatal period.

Improved hygiene has altered microbial exposure early in life, which has been suggested as a cause for the rising prevalence of atopic diseases [15]. Epidemiologic studies have shown that in particular food-borne and orofecal infections might be essential in the prevention of atopy [16]. It would seem unlikely however, that a stimulus that is potentially harmful to the host should be necessary for the postnatal maturation of a balanced immune system. Since the host's major and primary microbial stimulation occurs along with the establishment of the gut microflora, it has been suggested that exposure to commensal microflora or specific strains may represent a key modulator of the immune system against atopic diseases [17].

Our study shows that early colonisation with *Bacteroides fragilis *at age 3 weeks was associated with a positive API at age 3 years. No studies have looked prospectively at the relationship between early colonisation and development of asthma and particularly at API as outcome parameter. In a cross sectional study differences in gut flora have been observed between allergic and non-allergic infants [18]. In 62 Swedish and Estonian children counts of *Staphylococcus aureus *were higher and the proportion of *Bacteroides *was lower in the allergic children compared to the non-allergic children at age 2 years [18]. In a later prospective study Björksten followed 24 Estonian and 20 Swedish children from birth till 2 years of age. Infants who developed allergy, as demonstrated by skin prick test, were less often colonized with *Enterococcus *during the first month of life and had higher count of *Clostridium *at 3 months [19]. The prevalence of *Staphylococcus aureus *was also higher at 6 months, whereas the *Bacteroides *counts were lower at 12 months [19]. In a prospective study during the first 6 months of life Kalliomaki showed that differences in neonatal gut flora preceded the development of a positive skin prick test: allergic infants had more *Clostridium *and tended to have fewer *Bifidobacterium *at age 3 weeks in their stools than non allergic infants [5]. The fact that other authors did find differences in other intestinal bacterial species besides *Bacteroides *might be explained by the fact that timing of the sampling was different (older children), patient selection was different (allergic, skin test positive patients) or because the culture media used were less selective. Especially for *Bifidobacterium *(MTPY versus NPNL or RB medium), *Bacteroides *(BBE versus Schaedler medium including vancomycin and nalidixic acid) and *Lactobacillus *(LAMVAB versus MRS or Rogosa medium) we used more selective media than previous authors.

In a mouse model Mazmanian et al. showed that Polysaccharide A (PSA), a bacterial polysaccharide, stimulates the expansion of CD4^+ ^T cells and Th1 cytokine production [20]. This is not in agreement with the findings in our study. Several factors can explain this difference. First, a dose effect might be involved: we observed a median faecal *Bacteroides *count in infants of 3.5 log CFU/g (range 0–8.2) in contrast to the much higher counts during the monocolonisation of the germfree mice (> 10 log CFU/g). Similarly to endotoxin which has been shown to exert a Th1 response at high dose and a Th2 response at low dose, the cytokine response to PSA might be dose dependent. Second, PSA is only one of 8 capsular polysaccharides known to be produced by *Bacteroides fragilis *and regulated by DNA inversions. The immune response to these other 7 polysaccharides remains unknown. Moreover, Coyne et al. showed that fucosylation of these polysaccharides by the host results in rendering these bacteria immunological inert [21]. The fucosylation by the host was shown to be important in the gut colonisation ability of *Bacteroides fragilis*. We believe that intestinal *Bacteroides *species might be able to induce a Th2 cytokine response through binding of a TLR2 (Toll-like receptor) present on intestinal dendritic cells. Netea et al. showed that *Bacteroides *species stimulate cytokine release through TLR2-dependent (not TLR4) mechanisms [22]. TLR2 agonists induce a Th2 response by suppressing IL-12 production [23].

To our knowledge there are no data linking *Bacteroides *and asthma. However, two reports found an association between *Bacteroides *and IgE or IgG immune response in allergic children. Fukuda studied 867 junior high school children and administered a questionnaire concerning allergic symptoms. A higher IgG titer to *Bacteroides vulgaris *was found in the children with allergic symptoms [24]. In infants intolerant to extensively hydrolysed whey formula serum total IgE concentration was shown to correlate with faecal *Bacteroides *counts at age 4–6 months and bifidobacterial supplementation prevented increases in *Bacteroides *during weaning [25]. In non-allergic Estonian children the faecal counts of *Bacteroides *at age 5 years correlated positively with the serum IgE concentration [26]. One report in a mouse model supports the findings of our study. In BALB/c mice at 3 weeks of age a kanamycine induced elevation of serum IgE levels was enhanced by the inoculation with *Bacteroides vulgatus *[27]. Finally, a study in adults with pollen allergy showed an increased ratio of faecal counts of *Bacteroides fragilis *to *Bifidobacterium *during pollen season. This rise was prevented by bifidobacterial supplementation. In vitro, using peripheral blood mononuclear cells of these patients they also demonstrated that *Bacteroides fragilis *strains induced more Th2 cytokines but fewer Th1 cytokines compared with *Bifidobacterium *strains [28]. A limitation of the present study is the fact that no stool sample of the mother was included, so we cannot make any statement on the origin of the *Bacteroides fragilis *strains recovered in these infants. Finally, as in every longitudinal study, missing data is a hindrance. However, after multiple imputation, the interpretation of the original results has been confirmed.

## Conclusion

Modulation of the composition of the gut flora could contribute to alleviation of the symptoms of asthma. The desirable modification was identified as restraining of the *Bacteroides fragilis *flora. Detailed analyses of the gut flora of wheezing infants at different ages and at the species level are needed for a better understanding of the significance of the intestinal flora associated abnormalities in asthma. Such studies should also provide the means for more accurate targeting of treatment and prevention and thus more effective and better controlled modulation of the microbial milieu.

## Abbreviations

API: Asthma Predictive Index; BBE: Bacteroides Bile Esculine; CFU: Colony Forming Unit; GALT: Gut Associated Lymphoid Tissue; TLR: Toll-like receptor.

## Competing interests

The authors declare that they have no competing interests.

## Authors' contributions

CV was involved in the study design and concept, helped to draft and revise the manuscript and performed the statistical analysis. VN assisted in the data acquisition and helped revising the manuscript. SV assisted in the statistical analysis and helped revising the manuscript. HG assisted in the data acquisition and helped revising the manuscript. KD was involved in the study design and concept and helped to revise the manuscript. All authors read and approved the final manuscript.

## Pre-publication history

The pre-publication history for this paper can be accessed here:



## References

[B1] Martinez FD, Wright AL, Taussig LM, Holberg CJ, Halonen M, Morgan WJ (1995). Asthma and wheezing in the first six years of life. The Group Health Medical Associates. N Engl J Med.

[B2] Strachan DP (1989). Hay fever, hygiene, and household size. BMJ.

[B3] Murch SH (2000). The immunologic basis for intestinal food allergy. Curr Opin Gastroenterol.

[B4] Miettinen M, Lehtonen A, Julkunen I, Matikainen S (2000). Lactobacilli and Streptococci activate NF-kappa B and STAT signaling pathways in human macrophages. J Immunol.

[B5] Kalliomaki M, Kirjavainen P, Eerola E, Kero P, Salminen S, Isolauri E (2001). Distinct patterns of neonatal gut microflora in infants in whom atopy was and was not developing. J Allergy Clin Immunol.

[B6] Ouwehand AC, Isolauri E, He F, Hashimoto H, Benno Y, Salminen S (2001). Differences in Bifidobacterium flora composition in allergic and healthy infants. J Allergy Clin Immunol.

[B7] Pearce N, Weiland S, Keil U, Langridge P, Anderson HR, Strachan D, Bauman A, Young L, Gluyas P, Ruffin D (1993). Self-reported prevalence of asthma symptoms in children in Australia, England, Germany and New Zealand: an international comparison using the ISAAC protocol. Eur Respir J.

[B8] Castro-Rodriguez JA, Holberg CJ, Wright AL, Martinez FD (2000). A Clinical Index to Define Risk of Asthma in Young Children with Recurrent Wheezing. Am J Respir Crit Care.

[B9] Hartemink R, Rombouts FM (1999). Comparison of media for the detection of bifidobacteria, lactobacilli and total anaerobes from faecal samples. J Microbiol Methods.

[B10] Livingston SJ, Kominos SD, Yee RB (1978). New medium for selection and presumptive identification of the Bacteroides fragilis group. J Clin Microbiol.

[B11] Rada V, Petr J (2000). A new selective medium for the isolation of glucose non-fermenting bifidobacteria from hen caeca. J Microbiol Methods.

[B12] Hartemink R, Domenech VR, Rombouts FM (1997). LAMVAB – a new selective medium for the isolation of lactobacilli from faeces. J Microbiol Methods.

[B13] Molenberghs G, Verbeke G (2005). Models for Discrete Longitudinal Data.

[B14] Sudo N, Sawamura S, Tanaka K, Aiba Y, Kubo C, Koga Y (1997). The requirement of intestinal bacterial flora for the development of an IgE production system fully susceptible to oral tolerance induction. J Immunol.

[B15] Martinez FD, Holt PG (1999). Role of microbial burden in aetiology of allergy and asthma. Lancet.

[B16] Matricardi PM, Rosmini F, Ferrigno L, Nisini R, Rapicetta M, Chionne P, Stroffolini T, Pasquini P, D'Amelio R (1997). Cross sectional retrospective study of prevalence of atopy among Italian military students with antibodies against hepatitis A virus. BMJ.

[B17] Bjorksten B (1997). The environment and sensitisation to allergens in early childhood. Pediatr Allergy Immunol.

[B18] Bjorksten B, Naaber P, Sepp E, Mikelsaar M (1999). The intestinal microflora in allergic Estonian and Swedish 2-year-old children. Clin Exp Allergy.

[B19] Bjorksten B, Sepp E, Julge K, Voor T, Mikelsaar M (2001). Allergy development and the intestinal microflora during the first year of life. J Allergy Clin Immunol.

[B20] Mazmanian SK, Liu CH, Tzianabos AO, Kasper DL (2005). An immunomodulatory molecule of symbiotic bacteria directs maturation of the host immune system. Cell.

[B21] Coyne MJ, Reinap B, Lee MM, Comstock LE (2005). Human symbionts use a host-like pathway for surface fucosylation. Science.

[B22] Netea MG, Kullberg BJ, de Jong DJ, Franke B, Sprong T, Naber TH, Drenth JP, Meer JW van der (2004). NOD2 mediates anti-inflammatory signals induced by TLR2 ligands: implications for Crohn's disease. Eur J Immunol.

[B23] Agrawal S, Agrawal A, Doughty B, Gerwitz A, Blenis J, Van Dyke T, Pulendran B (2003). Cutting edge: different Toll-like receptor agonists instruct dendritic cells to induce distinct Th responses via differential modulation of extracellular signal-regulated kinase-mitogen-activated protein kinase and c-Fos. J Immunol.

[B24] Fukuda S, Ishikawa H, Koga Y, Aiba Y, Nakashima K, Cheng L, Shirakawa T (2004). Allergic symptoms and microflora in schoolchildren. J Adolesc Health.

[B25] Kirjavainen PV, Arvola T, Salminen SJ, Isolauri E (2002). Aberrant composition of gut microbiota of allergic infants: a target of bifidobacterial therapy at weaning?. Gut.

[B26] Sepp E, Julge K, Mikelsaar M, Bjorksten B (2005). Intestinal microbiota and immunoglobulin E responses in 5-year-old Estonian children. Clin Exp Allergy.

[B27] Sudo N, Yu XN, Aiba Y, Oyama N, Sonoda J, Koga Y, Kubo C (2002). An oral introduction of intestinal bacteria prevents the development of a long-term Th2-skewed immunological memory induced by neonatal antibiotic treatment in mice. Clin Exp Allergy.

[B28] Odamaki T, Xiao JZ, Iwabuchi N, Sakamoto M, Takahashi N, Kondo S, Iwatsuki K, Kokubo S, Togashi H, Enomoto T, Benno Y (2007). Fluctuation of fecal microbiota in individuals with Japanese cedar pollinosis during the pollen season and influence of probiotic intake. J Investig Allergol Clin Immunol.

